# Optimal localization strategies for non-palpable breast cancers –A network meta-analysis of randomized controlled trials

**DOI:** 10.1016/j.breast.2022.02.004

**Published:** 2022-02-08

**Authors:** Matthew G. Davey, John P.M. O'Donnell, Michael R. Boland, Éanna J. Ryan, Stewart R. Walsh, Michael J. Kerin, Aoife J. Lowery

**Affiliations:** Department of Surgery, The Lambe Institute for Translational Research, National University of Ireland, Galway, Galway, H91 YR71, Ireland

**Keywords:** Breast cancer, Localization, Precision oncology, Targeted surgery

## Abstract

**Purpose:**

Mammographic screening programmes have increased detection rates of non-palpable breast cancers. In these cases, wire-guided localization (WGL) is the most common approach used to guide breast conserving surgery (BCS). Several RCTs have compared WGL to a range of novel localization techniques. We aimed to perform a network meta-analysis (NMA) of randomized controlled trials (RCTs) comparing methods of non-palpable breast cancer localization.

**Methods:**

A NMA was performed according to PRISMA-NMA guidelines. Analysis was performed using R packages and Shiny.

**Results:**

24 RCTs assessing 9 tumour localization methods in 4236 breasts were included. Margin positivity and reoperation rates were 16.9% (714/4236) and 14.3% (409/2870) respectively. Cryo-assisted localization had the highest margin positivity (28.2%, 58/206) and reoperation (18.9%, 39/206) rates. Compared to WGL (n = 2045 from 24 RCTs) only ultrasound guided localization (USGL) (n = 316 from 3 RCTs) significantly lowered margin positivity (odds ratio (OR): 0.192, 95% confidence interval (CI): 0.079–0.450) and reoperation rates (OR: 0.182, 95%CI: 0.069–0.434). Anchor-guided localization (n = 52, 1 RCT) significantly lowered margin positivity (OR: 0.229, 95%CI: 0.050–0.938) and magnetic-marker localization improved patient satisfaction (OR: 0.021, 95%CI: 0.001–0.548). There was no difference in operation duration, overall complications, haematoma, seroma, surgical site infection rates, or specimen size/vol/wt between methods.

**Conclusion:**

USGL and AGL are non-inferior to WGL for the localization of non-palpable breast cancers. The reported data suggests that these techniques confer reduced margin positivity rates and requirement for re-operation. However, caution when interpreting results relating to RCTs with small sample sizes and further validation is required in larger prospective, randomized studies.

## Highlights

• Ultrasound-guided (USGL) and anchor-guided (AGL) localization had optimal outcomes.
• These methods significantly lowered margin positivity (odds ratio: 0.192 & 0.229).
• However, small sample sizes in trials evaluating USGL and AGL limit these results.
• Operation duration, complications, or specimen data were comparable for all methods.

## 1 Introduction

The establishment of mammographic breast cancer screening programmes and enhancement of diagnostic strategies for breast cancer have facilitated an increase in the detection of non-palpable breast lesions [1]. In the majority of such cases, breast conserving surgery (BCS) is indicated, and wire-guided localization (WGL) is currently the most widely used, standardized technique to guide tumour excision [2]. First described by Dodd et al., in 1965 [3], WGL involves the use of preoperative ultrasound (US) or stereotactic guidance to localize the tumour and the insertion of a self-retaining wire into the lesion, which is then used intraoperatively by the surgeon to guide resection [4]. Accurate localization of the lesion is crucial to ensure tumour margins are clear of disease, to minimise the requirement for reoperation, and to facilitate successful BCS. Unfortunately, WGL of non-palpable cancers have been reported to have positive margin rates as high as 17.0%, often requiring reoperation, and conferring an increased risk of local recurrence [5,6].

Although WGL is currently the most common approach used to guide the localization of non-palpable breast cancer, there are a number of limitations to be considered with this approach: WGL may be complicated by the displacement of the guide-wire prior to or during the operation, and inaccurate placement of the wire in relation to the tumour may negatively impact clear margin rates. The overall process of wire insertion is time-consuming and there are logistical challenges associated with this approach, including an additional procedure which depends upon mammography or ultrasound to aid insertion and is typically performed on the morning of surgery in the radiology department, often adding complexity to theatre scheduling. Moreover, patient reported outcomes of WGL include discomfort and pain [7,8]. As previously outlined, WGL is associated with margin positivity rates of up to 17%, which require reoperation (to ensure disease clearance), and additional cost [9]. Finally, the wire acts simply as a guide for the operating surgeon. This indicates that with the aid of pre-operative imaging, a judicial approximation of the extent of the entire tumour volume is made intraoperatively, which may result in human error. Thus, efforts to enhance breast cancer localization are imperative in the current breast cancer surgery paradigm.

There are several novel approaches used to localize non-palpable breast tumours [10,11]: Radioactive occult lesion localization (ROLL), which involves intratumoral injection of ^99^Technetium into the tumour under ultrasound-guidance with intraoperative use of a handheld gamma probe to localize the extent of the tumour [12]; Radioactive seed localization (RSL) which involves the introduction of small, radio-opaque, ^125^Iodine-labelled titanium pellets into the centre of the breast lesion to guide resection [13]; intraoperative ultrasound-guided localization (USGL), which involves the real-time localization of breast tumours intraoperatively by either the surgeon or a radiologist to guide resection [14], intraoperative supine magnetic resonance imaging (SMRI), which uses real-time magnetic resonance imaging to quantify the location, deformation, and potential displacement of the tumour while the patient is supine on the operating table [15], anchor-guided localization (AGL), which involves use of a device with a calibrated shaft capable of extending fixation wires 3 cm radially to secure the target lesion in place before transfer to the operating room [16], cryo-assisted localization (CAL), which involves using a cryoprobe (under ultrasound- or mammographic-guidance) to create an ice margin around the breast lesion in order to make the lesion palpable to facilitate resection [17,18], indocyanine green fluorescence-guided localization (IL), which involves injection of a fluorescent dye into the longest axis (under ultrasound-guidance) of the tumour before a photodynamic eye is used to localize the tumour intra-operatively [19], and finally, magnetic-marker localization (ML), which involves the magnetic tracing of a non-ferromagnetic marker coil using a magnetic localizer intraoperatively [20]. Similar to ROLL and RSL, ML involves preoperative placement of the ‘target’ (i.e.: a magnetic marker coil) into the breast lesion under ultrasound- or mammographic-guidance [21]. Additionally, radiofrequency-guided localization and radar reflectors have been introduced as novel methods of localizing non-palpable breast lesions [22,23].

Recent studies suggest these novel methods of tumour localization may be effective in reducing margin positivity, the requirement for reoperation, and complication rates among patients undergoing BCS for non-palpable breast tumours [[24], [25], [26], [27]], while other studies report moderate results compared to WGL [28]. Thus, analyses comparing such strategies may prove valuable in enhancing clinical and oncological outcomes for prospective patients being treated for non-palpable breast cancer.

Several meta-analyses have been performed comparing these single novel approaches of breast tumour localization to WGL [7,[29], [30], [31]]. However, the data from studies assessing the most effective means of localizing non-palpable breast cancers remains sparce and inconsistent, with no single optimal localization means identified. Therefore, the advantage of using network meta-analysis (NMA) methodology employed here is that it allows simultaneous comparison (both direct and indirect) of more than two treatments [32,33]. Accordingly, the aim of the current study was to perform and updated systematic review and NMA of RCTs which comprehensively evaluated all data comparing methods of localizing non-palpable breast cancers for BCS.

## 2 Methods

A systematic review was performed in accordance to the ‘Preferred Reporting Items for Systematic Reviews and Meta-Analyses’ (or PRISMA) extension statement for reporting of systematic reviews incorporating network meta-analyses of healthcare interventions [34]. Local institutional ethical approval was not required as all data used in this analysis was obtained from a previously published resource. This study was prospectively registered with the International Prospective Register of Systematic Reviews (PROSPERO -CRD42021286784).

### 2.1 Study eligibility

All published RCTs with full-text manuscripts comparing the outcomes of at least two means of localizing non-palpable cancers in the breast were included. Studies included those evaluating localization techniques for suspected invasive or non-invasive breast cancers and were not limited based on patient demographics, tumour histopathological features or tumour molecular subtyping. Studies reporting outcomes following localization of invasive cancers which had associated non-invasive disease were included. Included studies were expected to report on the primary outcome of interest. Studies comparing localization strategies for sentinel lymph node biopsy (SLNB), including data from patients with advanced breast cancer, and those not published in the English language were excluded. Included studies were not restricted by year of publication.

### 2.2 Population, intervention, comparison, outcomes (PICO)

Using the PICO framework [35], the aspects the authors wished to address were:

Population –Patients with a newly diagnosed non-palpable breast cancer aged 18 years or older who were due to undergo tumour localization prior to BCS.

Intervention – Any patient who was having their tumour localised using any technique, other than WGL.

Comparison – Any patient who was having their tumour localised using WGL.

Outcomes – The primary outcome of interest was:

• Margin positivity rates following resection of a non-palpable breast cancer. Margin widths included any width reported in each study.

The secondary outcomes of interest included:

• Rates of reoperation rate following initial procedure for a non-palpable breast cancer. These included re-excision of margins, a further wide-local excision and completion mastectomy, and excluded surgical management of local recurrences.
• Timing/duration of operation, defined as the time taken in minutes from opening skin to skin closure.
• Rates of complications, including and not limited to surgical site infections (SSIs), seroma, bleeding or haematoma.
• Satisfaction rates among patients and surgeons, expressed as dichotomous variables (i.e. satisfied or unsatisfied).
• Cost of each localization technique (in euros). In cases where cost was provided in another currency, local conversion rates were applied for comparability.
• Specimen characteristics such as specimen volume (centimetres^3^), specimen size (diameter in centimetres), and specimen weight (grams) following resection.
• Disease recurrence, defined as locoregional and/or distant recurrence of the primary cancer following resection with clear margins.

### 2.3 Search strategy

A formal systematic search of the *PubMed, Scopus* and *Cochrane Central Register of Controlled Trials* electronic databases was performed for relevant titles. This search was performed by two independent reviewers (MGD & JPMO’D), using a predetermined search strategy that was designed by the senior authors. This search included the search terms: (breast cancer), (non-palpable), (breast conserving surgery) linked using the Boolean operator ‘AND’. Manual cross-referencing of reference lists from previous systematic reviews, meta-analyses and included trials was undertaken.

Manual removal of duplicate studies was performed before all titles were screened. Thereafter, RCTs considered to be appropriate had their abstracts and/or full text reviewed. Retrieved studies were reviewed to ensure inclusion criteria were met for the primary outcome at a minimum, with discordances in opinion resolved through consultation with the senior author (AJL). Data extraction was also performed by two independent reviewers (MGD & JPMO’D), with study details, basic patient clinicopathological characteristics and surgical data all recorded. The final search was performed on the 22^nd^ September 2021.

### 2.4 Data management and analysis

Descriptive statistics were used to outline characteristics of included trials. Rates of tumour margin positivity, requirement for reoperation and complications were expressed as dichotomous or binary outcomes, reported as odds ratios (ORs) were expressed with 95% confidence intervals (CIs). ORs were calculated, using crude event RCT data, to compare interventions using per-protocol data, where applicable. Comparative operation time were calculated using mean values, standard deviations (SD) and pooled mean variance. WGL was the principal comparator for all analyses.

Bayesian NMAs were conducted using netameta and Shiny packages for R [36]. Effect sizes were described with a 95% CI. Results were considered statistically significant at the *P* < 0.050 level if the 95% CI did not include the value of one. Estimates of mean and SDs were calculated using standard statistical methods, where applicable [37,38]. Rank probabilities were plotted against the possible ranks for all competing treatments. The confidence in estimates of the outcome was assessed using ‘Confidence in Network Meta-Analysis’ (CINeMA) [39]. Methodological assessment of included studies was undertaken using the Cochrane Risk of Bias assessment tool [40].

## 3 Results

### 3.1 Literature search and study characteristics

**Fig. 1:**
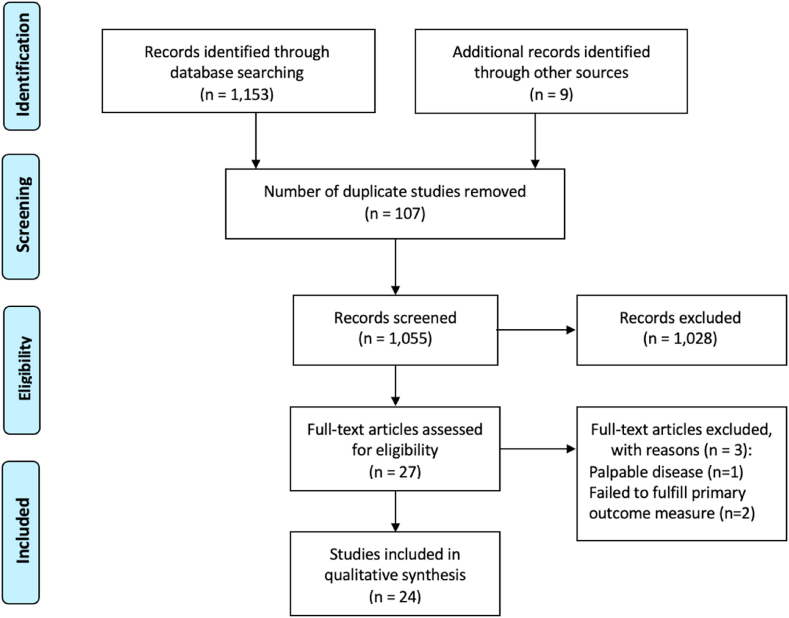
PRISMA flow diagram detailing the systematic search process.

The systematic search strategy identified a total of 1162 studies, of which 107 duplicate studies were manually removed. The remaining 1055 studies were screened for relevance, before 27 full texts were reviewed. In total, 24 RCTs fulfilled our inclusion criteria and were included in this systematic review and NMAs (Fig. 1) [16,27,[41], [42], [43], [44], [45], [46], [47], [48], [49], [50], [51], [52], [53], [54], [55], [56], [57], [58], [59], [60], [61], [62]]. Of the 24 RCTs included in this analysis, almost 40.0% were conducted in European surgical research institutions (37.5%, 9/24). Publication dates ranged from 2001 to 2021 (Table 1).

**Table 1:** Data from the 24 included randomized controlled trials included in this systematic review and network meta-analysis.

Author	Year	Country	Trial Number	Total (N)	Breast (N)	Intervention (vs. WGL)
Postma	2012	Netherlands	NCT00539474	314	316	ROLL
Duarte	2016	Columbia	C41030610-019	129	129	ROLL
Medina-Franco	2008	Mexico	–	100	100	ROLL
Rampaul	2004	UK	–	93	93	ROLL
Martinez	2009	Spain	–	134	134	ROLL
Ocal	2011	Turkey	–	108	108	ROLL
Moreno	2008	Brazil	–	120	120	ROLL
Kanat	2016	Turkey	–	36	36	ROLL
Alikhassi*	2016	Iran	–	60	60	ROLL
Tang	2011	China	–	157	157	ROLL + Dye
Langhans	2017	Denmark	R72-A4701–13-S9	409	413	RSL
Taylor	2021	Australia	ACTRN12613000655741	659	664	RSL
Bloomquist	2015	USA	–	125	125	RSL
Lovrics	2011	Canada	NCT00225927	305	305	RSL
Parvez	2014	Canada	–	73	73	RSL
Gray	2001	USA	–	97	97	RSL
Rahusen	2002	Netherlands	–	49	49	USGL
Hoffman	2019	Germany	NCT02222675	47	47	USGL
Hu	2020	China	–	520	520	USGL
Tafra	2016	USA	–	320	320	CAL
Struik	2021	Netherlands	NL6553	67	67	ML
Tong	2019	China	–	62	62	IL
Israel	2002	USA	–	114	114	AGL
Barth Jr.	2019	USA	NCT01929395	137	137	SMRI

N; Number, WGL; wire-guided localization, ROLL; radio-guided occult lesion localization, RSL; radioactive seed localization, USGL; ultrasound-guided localization, CAL; cryo-assisted localization, ML; magnetic-marker localization, IL; indocyanine green fluorescence-guided lumpectomy, AGL; anchor-guided localization.

SMRI; intraoperative supine magnetic resonance imaging, UK; United Kingdom, USA United States of America.

*Incalculable at meta-analyses results reporting no positive results for the primary outcome of interest.

### 3.2 Clinicopathological and surgical characteristics

In total, there was data included from 4225 patients (4236 breasts) with mean age at diagnosis of 56.6 years (range 25–81 years). Overall, 63.1% of BCS surgery was performed for invasive disease (1614/2559). Additional available clinicopathological data of patients included in the 24 RCTs are outlined in Supplementary Appendix 1.A.

In total, 48.3% of patients underwent WGL (2045/4236–24 RCTs), 20.0% underwent RSL (845/4236–6 RCTs), 15.1% underwent ROLL (640/4236–10 RCTs), 1.6% underwent intraoperative SMRI (68/4236 - 1 RCT), 7.5% underwent USGL (316/4236–3 RCTs), 0.8% underwent ML and IL (both 32/3,4,236–1 RCT) respectively, 4.9% underwent CAL (206/4236–1 RCT), and 1.2% underwent AGL (52/4236–1 RCT) (Table 2). Overall, 98.0% of patients underwent SLNB (1530/1562–7 studies).

**Table 2:** Surgical data for all methods of breast tumour localization.

Intervention (Number of Trials)	Number (%)	Positive margins (%)	Reoperation rates (%)	Operation Time in minutes (SD)	Complications (%)	Haematoma (%)	Seroma (%)	SSI (%)
WGL (24)	2045 (48.3%)	411 (20.1%)	239/1379 (17.3%)	42.1 (13.2)	59/842 (7.0%)	11/594 (1.9%)	14/511 (2.7%)	20/522 (3.8%)
USG (3)	316 (7.5%)	17 (5.4%)	14/289 (4.8%)	73.0 (17.5)	–	–	–	–
ROLL (10)	640 (15.1%)	110 (17.2%)	37/379 (9.8%)	28.0 (9.7)	24/478 (4.6%)	11/384 (2.9%)	7/283 (2.5%)	13/387 (3.4%)
RSL (6)	845 (20.0%)	99 (11.7%)	71/689 (10.3%)	19.0 (9.0)	31/359 (8.6%)	6/163 (3.7%)	5/207 (2.4%)	6/207 (2.9%)
ML (1)	32 (0.8%)	2 (6.3%)	1/32 (3.1%)	54.0 (14)	4/32 (12.5%)	1/32 (3.1%)	2/32 (6.3%)	1/32 (3.1%)
IL (1)	32 (0.8%)	4 (12.5%)	–	31.0 (5.0)	–	–	–	–
CAL (1)	206 (4.9%)	58 (28.2%)	39/206 (18.9%)	31.0				
AGL (1)	52 (1.2%)	5 (9.6%)	5/52 (9.6%)	9.7				
SMRI (1)	68 (1.6%)	8 (11.8%)	8/68 (11.8%)	–	–	–	–	–
Total (24)	4236 (100.0%)	714 (16.9%)	409/2870 (14.3%)	Mean: 38.4 (12.3)	116/1711 (6.8%)	25/1217 (2.1%)	28/1033 (2.7%)	40/1226 (3.3%)

SD; standard deviation, SSI; surgical site infection, WGL; wire-guided localization, USGL; ultrasound-guided localization, ROLL; radio-guided occult lesion localization, RSL; radioactive seed localization, ML; magnetic-marker localization, IL; indocyanine green fluorescence-guided lumpectomy, CAL; cryo-assisted localization, AGL; anchor-guided localization, SMRI; intraoperative supine magnetic resonance imaging.

### 3.3 Margin positivity

**Fig. 2:**
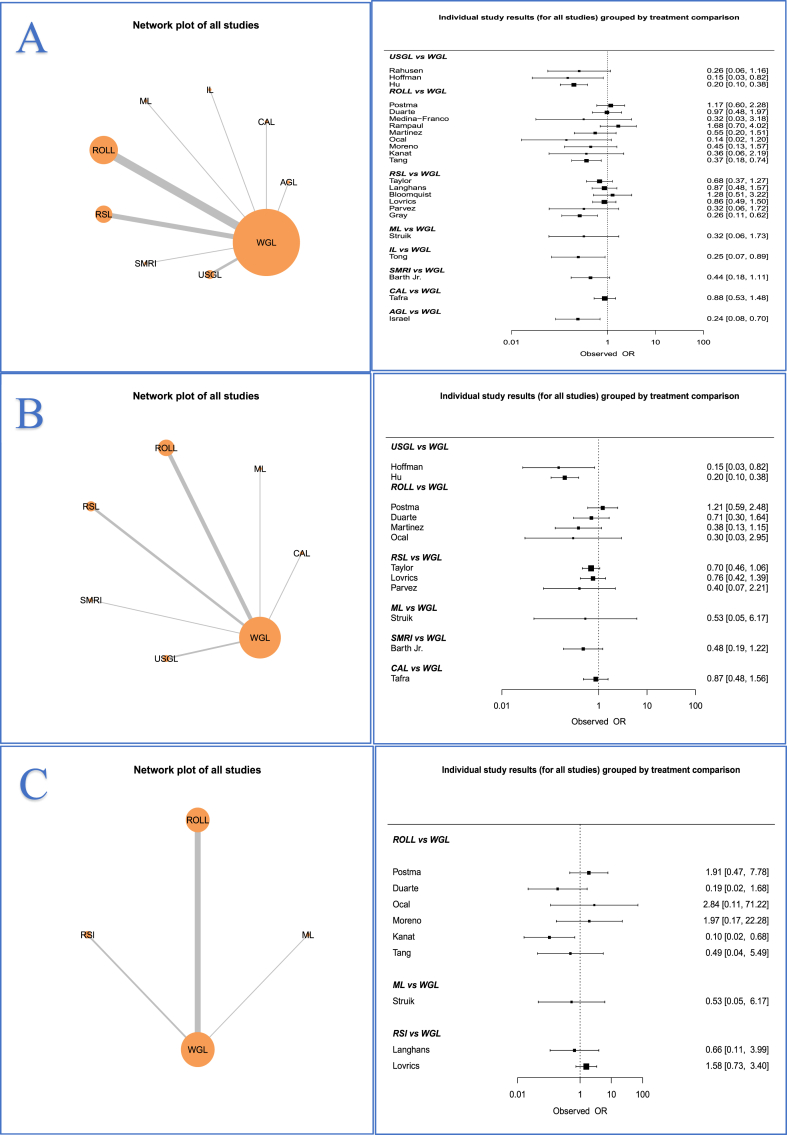
Network plots and data summary of all included individual studies assessing rates of (A) margin positivity, (B) reoperation rates, and (C) complications, odds ratios and 95% confidence intervals.

**Fig. 3:**
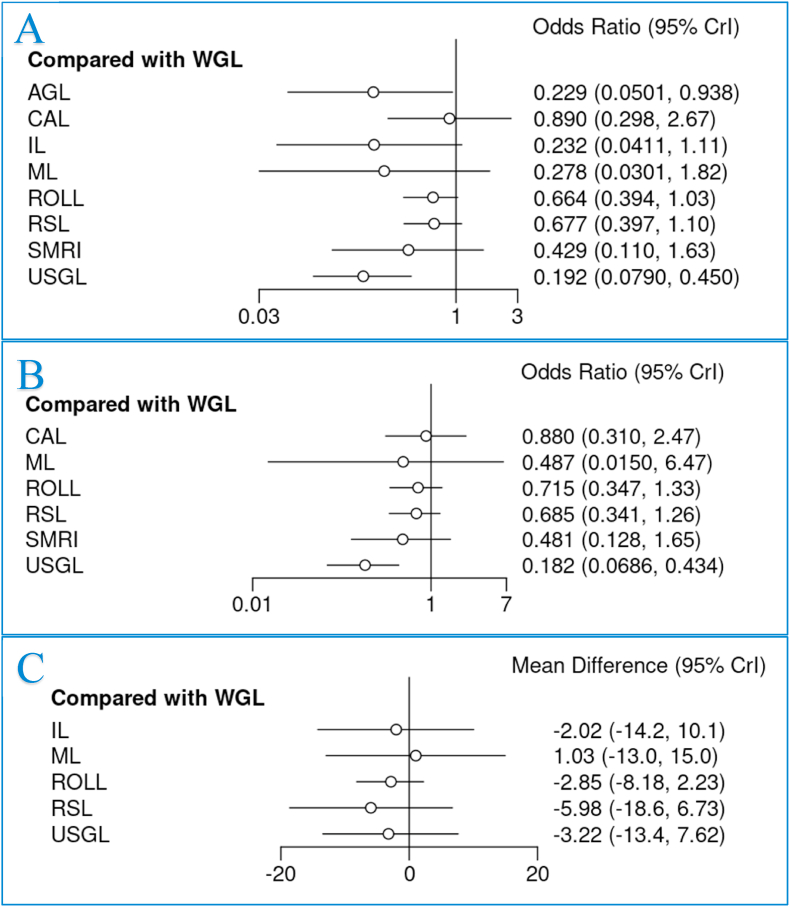
Forest plots comparing wire-guided localization (WGL) to all other interventions for (A) margin positivity, (B) reoperation rates, and (C) operation duration.

All 24 RCTs reported outcomes for analysis in relation to margin positivity (100.0%) (Fig. 2A). Overall, the margin positivity rate was 16.9% (714/4236). CAL had the highest associated margin positivity rate (28.2%, 58/206), followed by WGL (20.1%, 411/2045), while USGL had the lowest margin positivity rates (5.4%, 17/316) (Table 2). When compared with WGL, both USGL (OR: 0.192, 95% CI: 0.079–0.450) and AGL (OR: 0.229, 95% CI: 0.050–0.938) had significantly lower margin positivity rates. In this NMA, there was no significant difference in margin positivity rates for patients undergoing IL (12.5%, 4/32), ML (6.3%, 2/32), ROLL (15.1%, 110/640), RSL (11.7%, 99/845), CAL (28.2%, 58/206) or SMRI (11.8%, 8/68) (Fig. 3A). Ranking tables and plots for interventions and margin positivity rates are presented in Supplementary Appendix 1.B. Definitions for margin status from each of the 24 RCTs are outlined in Supplementary Appendix 1.C.

### 3.4 Reoperation rate

Thirteen RCTs reported reoperation rates (54.2%) (Fig. 2B) with 14.3% of cases required reoperation (409/2879) overall. CAL (18.9%, 39/206) had the highest reoperation rate, followed by WGL (17.3%, 239/1379) (Table 2). When compared with WGL, USGL had significantly lower reoperation rates (OR: 0.182, 95% CI: 0.069–0.434). None of the other methods of breast tumour localization significantly reduced the requirement for reoperation on NMA (Fig. 3B). Ranking tables and plots for interventions and the requirement for reoperation are presented in Supplementary Appendix 1.D.

### 3.5 Operation time

Overall, 14 RCTs reported operation times. The mean combined time of tumour localization and BCS was 38.4 min (SD: 12.3 min) (Table 2). Despite USGL having the longest mean operation time (73.0 min, SD: 17.5 min), there was no significant difference observed in operation time for any of the methods of breast tumour localization (Fig. 3C). Ranking tables and plots for interventions and the operation time are presented in Supplementary Appendix 1.E.

Treatment effect strategies are outlined for (A) margin positivity, (B) the requirement for reoperation, and (C) duration of operation in Supplementary Appendix 1.F.

### 3.6 Overall complication rates

**Fig. 4:**
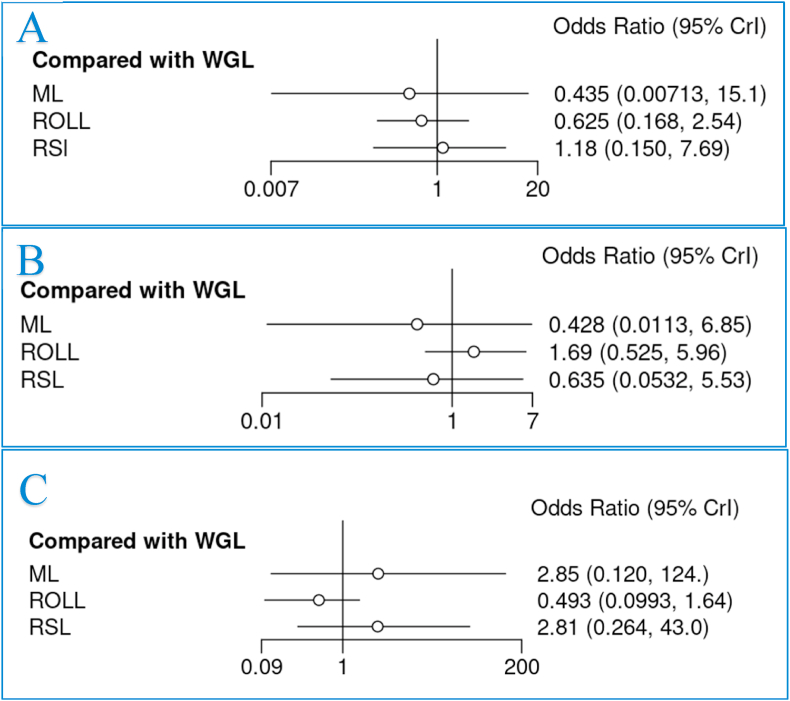
Forest plots comparing wire-guided localization (WGL) to magnetic-marker localization (ML), radio-guided occult lesion localization (ROLL) and radioactive seed localization (RSL) for (A) overall complications, (B) haematoma, and (C) seroma.

As illustrated network plot 3, 10 RCTs reported overall complication rates following breast tumour localization (41.7%) (Fig. 2C). The overall complication rate was 6.8% (116/1711), with patients undergoing ML localization having the highest complication rate (12.5%, 4/32) (Table 2). None of the assessed methods of breast tumour localization significantly reduced the overall complication rates (Fig. 4A).

### 3.7 Haematoma, seroma and surgical site infection rates

Overall, 7 RCTs reported specific complications including haematoma, seroma and surgical site infection (SSI) rates following breast tumour localization (29.2%). In these studies, 2.1% and 3.2% of cases were complicated by haematoma (25/1217) and seroma respectively (28/887), while 3.3% had a SSI (40/1226) (Table 2). There was no difference in haematoma or seroma rates for each of the assessed methods of breast tumour localization (Fig. 4B-4C). Ranking tables and plots for overall complications, haematoma, seroma, and SSIs are presented in Supplementary Appendix 1.G-1.J. Treatment effect strategies are outlined for (A) overall complications, (B) haematoma, (C) seroma, and (D) surgical site infections in Supplementary Appendix 1.K.

### 3.8 Satisfaction rates

In total, 6 RCTs reported either patient or surgeon satisfaction with the localization strategies (25.0%). All 6 of these RCTs reported patient satisfaction rates (81.7%, 652/798). Patients undergoing ML had the highest satisfaction rates (100.0%, 32/32). In the meta-analysis, ML was associated with enhanced patient satisfaction when compared to WGL (OR: 50.600, 95% CI: 2.010–3.16e^3^) (Supplementary Appendix 1.L.A).

In the 2 RCTs reporting satisfaction rates among surgeons, the overall satisfaction rate was 75.0% (147/196) (Table 3). Surgeon satisfaction rates were highest for those undergoing ROLL (98.4%, 63/64). In the NMA, surgeon satisfaction rates were comparable for WGL, ML and ROLL (Supplementary Appendix 1.L.B). Data pertaining to pain and cosmesis are outlined in Supplementary Appendix 1.M.

**Table 3:** Histopathological and outcome data for all methods of breast tumour localization.

Intervention	Patient Satisfaction (%)	Surgeon Satisfaction (%)	Specimen size (mm)	Specimen volume (mm3)	Specimen Weight (g)	Cost (€)	Recurrence
WGL	306/397 (77.1%)	66/100 (66.0%)	48.0	65.0	36.8	163.40	–
USG	–	–	8.0	61.4	38.0	64.68	–
ROLL	286/338 (85.6%)	63/64 (98.4%)	51.3	39.9	31.1	181.83	–
RSL	28/35 (80.0%)	–	13.2	121.3	35.1	–	–
ML	32/32 (100.0%)	18/32 (56.3%)	13.9	39.5	36.0	–	–
IL	–	–	–	56.0	–	–	–
CAL	–	–	–	–	–	–	–
AGL	–	–	9.0	–	–	–	–
SMRI	–	–	–	74.0	–	–	–
Total(%)/Mean	652/798 (81.7%)	147/196 (75.0%)	47.2	60.4	35.4	136.13	–

WGL; wire-guided localization, USGL; ultrasound-guided localization, ROLL; radio-guided occult lesion localization, RSL; radioactive seed localization, ML; magnetic-marker localization, IL; indocyanine green fluorescence-guided lumpectomy, CAL; cryo-assisted localization, AGL; anchor-guided localization, SMRI; intraoperative supine magnetic resonance imaging.

### 3.9 Histopathological specimen characteristics

In total, 6, 11 and 10 RCTs reported outcomes in relation to specimen size, volume, and weight respectively. The mean specimen size was 47.0 mm. Specimens obtained following ROLL were the largest (mean size: 51.3 mm). The mean specimen volume was 60.4 cm^3^. Specimens obtained following RSL were the largest (mean volume: 121.3 cm^3^ and those undergoing ML were the smallest (mean volume: 39.5 cm^3^). The mean specimen weight was 35.3 g. Specimens obtained following USGL were the largest (mean weight: 38.0 g) and those undergoing ROLL were the smallest (31.1 g) (Table 3). At meta-analysis, we observed no difference in specimen size, volume, or weight (Supplementary Appendix 1.N.A-1.N.C).

### 3.10 Cost of tumour localization methods

Only 2 RCTs reported the cost benefit of tumour localization techniques. The mean costs of tumour localization reported were 136.13 euros. USGL was the least expensive method of tumour localization (mean cost: 64.68 euros), followed by WGL (mean cost: 163.41 euros), and then by ROLL (mean cost: 181.83 euros) (Table 3).

### 3.11 Breast cancer recurrence

None of the included RCTs reported outcomes in relation to tumour recurrence following non-palpable tumour localization for BCS (Table 3).

### 3.12 Publication bias

There was low to moderate risk of bias among the included RCTs: Overall, 10 of the included RCTs had low-risk of bias (41.7%) [27,42,45,50,51,53,54,57,61,62], 12 of the RCTs included some concerns for bias (60.0%) [16,24,41,44,[47], [48], [49],^52^,^55^,[58], [59], [60]], while 2 RCTs were considered high-risk of bias (10.0%) [46,56]. Risk of bias assessment is illustrated in the Supplementary Appendix 1.O.

## 4 Discussion

The breast cancer treatment paradigm has evolved such that both mammographic screening programs and more accurate diagnostics have facilitated earlier detection of breast cancers, which has translated into improved clinical and oncological outcomes. Consequently, the detection of small, non-palpable breast tumours requiring localization has increased, and WGL is currently the most widely used method of tumour localization. The most important finding in this NMA of 24 RCTs encompassing 4236 patients is the data illustrating the highest margin positivity (CAL: 28.2%, 39/206, WGL: 20.1%, 411/2045) and reoperation (CAL: 18.9%, 39/206, WGL: 17.3%, 239/1379) rates occurring in those undergoing CAL and WGL. Although the results of this analysis suggest overall margin positivity and reoperation rates are similar for conventional WGL and the 8 other novel localization methods, the crude numbers illustrate a difference in these outcome measures which is likely to be clinically relevant: We observed an absolute reduction of almost 75% in margin positivity for those undergoing USGL (USGL: 5.4% vs. WGL: 20.1%), as well as an estimated 80% estimated relative reduction in margin positivity (OR: 0.192) and the requirement for reoperation (OR: 0.182) respectively. Furthermore, using ML and AGL reduced margin positivity rates by greater than 50% relative to WGL (ML: 6.3% OR: 0.278, AGL: 9.6% OR: 0.229), although these results are limited by the availability of just one RCT evaluating these localization techniques respectively. Additionally, margin positivity rates were modest following RSL (11.7%, 99/845), IL (12.5%, 4/32) and SMRI (11.8%, 8/68). This is somewhat unsurprising as the displacement of the marker in strategies such as ML, RSL, and ROLL is rare once they are inserted [10], and the utility of these strategies is much more straight forward, intuitive and subject to less operator dependence/variation for the clinician [63]. This may contribute to the increased surgeon satisfaction rates observed in relation to ROLL (98.4% vs. 66.6% for WGL). Similarly, AGL provides direct localization of the tumour specimen preoperatively using fixation wiring, facilitating targeted surgery while limiting the risk of displacement [16], while IL provides coherent visualization of the tumour boundaries facilitating accurate resection [64]. In tandem, the combined raw data in relation to margin status and NMA results capture the clinical relevance of using these novel localization strategies in reducing margin positivity when performing breast conserving surgery for non-palpable cancers. Previous meta-analyses have been undertaken in attempt to address the most effective method of breast tumour localization, including a NMA of 18 RCTs performed by Athanasiou et al. which highlighted reduced margin positivity rates when using USGL (OR: 0.19), [25]. In a recent meta-analysis of RCTs, Kiruparan et al. outlined that WGL was associated with higher margin positivity rates than ROLL (OR: 1.520, 95% CI: 1.030–2.250) [40]. This result was not supported by the data in our NMA (OR: 0.771, 95% CI: 0.421–1.220), due to inclusion of an additional RCT by Tang et al. who report a margin positivity rate of 20.3% following combined ROLL and methylene dye (16/79) [59]. Wang et al. and Pouw et al. both previously performed meta-analyses including both retrospective and prospective studies comparing the value of RSL versus WGL respectively [7,28]. Overall, Wang et al. illustrated a significant advantage of using RSL in reducing positive margin rates (OR: 0.72, 95% CI: 0.56–0.92) and reoperation rates (OR: 0.68, 95% CI: 0.52–0.88), however these results were not fully replicated with respect to margins (OR: 0.85, 95% CI: 0.55–1.31), and reoperation (OR: 0.80, 95% CI: 0.48–1.32), when including RCT data only, which is keeping with our findings [7]. Pouw et al. report a margin positivity rate of 10.3% (281/2732) and re-excision rate of 14.2% (343/2415) following RSL, which is comparable to the results observed in our analysis (margin positivity: 11.7% (99/845), reoperation rate: 10.3% (71/689)) [28]. However, 50% of studies included in their analysis were retrospective in nature (8/16), while we only included data from RCTs. With respect to RSL, results from our analysis suggests RSL trends towards significance when compared to WGL for both margin positivity (OR: 0.677, 95% CI: 0.397–1.110) and reoperation rates (OR: 0.685, 95% CI: 0.341–1.260), when only RCTs are included. Garzotto et al. recently performed a meta-analysis illustrating the role of non-wired, non-ionizing techniques in enhancing surgical outcomes such as margin positivity and reoperation rates, however this analysis was limited to non-randomized studies [5]. Therefore, this NMA of RCTs holds the highest level of evidence assessing current strategies of breast tumour localization, compared to conventional WGL.

Despite observing significant differences in margin positivity and reoperation rates for USGL and AGL compared to WGL, there was no significant difference in specimen size, volume, or weight at meta-analysis for the 5 tumour localization strategies assessed for which this data was available (WGL, ROLL, RSL, SMRI and USGL). This is an interesting finding: USGL proved efficacious in enhancing oncological and surgical outcomes (i.e.: reducing margin positivity and reoperation rates) without significantly increasing the amount of tissue being resected at surgery. While 3 RCTs reported outcomes for USGL, we must acknowledge that there were 2 small trials with less than 100 patients overall [41,42], and the largest RCT using USGL excluded cases of invasive disease with associated DCIS [27]. DCIS is known to increase margin positivity rates [65], which therefore may have potentially biased outcome measures in favor of this localization strategy. Therefore, we must be cognizant of this when interpreting results in relation to USGL. USGL was also the most cost-effective localization technique in this systematic review (USGL: €64.68 versus mean: €136.13). Conversely, although there was no difference in operation times between localization modalities at meta-analysis (Fig. 3C), the raw data from the three studies reporting operation time in this study indicates that USGL had the longest mean operation time (USGL: 73.0 min versus Mean: 38.4 min) which may offset these anticipated cost savings. Of the 3 RCTs comparing USGL and WGL, just two reported operation duration and these were very similar for both localization strategies [41,42], and it is important to note that other important surgical factors (such as sentinel lymph node sampling, oncoplasty, etc.) potentially confound operative duration. However, this theory surrounding USGL prolonging operative duration may subsequently be challenged through the conceptualization that reduced margin and reoperation rates will indirectly reduce operating room costs in the long-term. Moreover, these reported prolonged operation times would be likely to decrease inversely as the learning curve for clinicians using intra-operative USGL improves. Overall, we must acknowledge that these results must be interpreted with caution, as surgical specimen data (i.e.: size, volume, weight, etc.) was not provided in all the 24 included RCTs. Furthermore, the raw data in this study indicates that the mean specimen volume for RSL was approximately three times larger than ROLL and ML, and twice as large as USGL, IL and WGL respectively.

Overall, satisfaction levels and complication rates did not differ greatly among tumour localization techniques in this meta-analysis. However, patients who had non-palpable tumours localized using ML reported increased satisfaction with ML localization (*P* = 0.032) and overall satisfaction with the procedure following BCS (OR: 50.600, 95% CI: 2.010–3.16e^+03^), supporting the routine use of ML as a reasonable alternative to WGL. Despite these promising results, it is important to highlight that 12.5% (4/32) of patients undergoing ML had a post-operative complication, illustrating there will inevitably be a learning curve for clinicians using these novel localization methods. Moreover, these results must be interpreted with caution, as just 32 patients in this NMA underwent ML, with further RCTs required to fully establish the risk profile and efficacy associated with ML overall. Similarly, caution must be taken with interpretating the results of the localization strategies with small sample sizes: Only 3 of the 24 studies randomized patients to USGL [27,41,42] and just 1 trial randomized patients to ML [45], IL [53], CAL [61], and AGL [16] respectively (all versus WGL). Moreover, the total number of randomized patients in some of these trials included less than 100 patients [41,42,45,53]. In total, just 2 patients had positive margins following ML (6.3%) [45] as did 5 patients undergoing AGL (9.6%). Although USGL may appear advantageous in this setting, well described disadvantages of breast ultrasound include operator variability, lack of standardization and difficulties identifying ductal carcinoma in-situ must be taken into consideration [[66], [67], [68]], Furthermore, included RCTs varied in that one involved both surgeons and radiologists localizing the lesion intraoperatively [41], while others relied solely upon surgeons to conduct the localization [27,42], likely explaining the prolonged operation times observed in this systematic review (USGL increased the mean duration of surgery to 73.0 min).

This NMA highlights that margin positivity and reoperation rates are the key primary outcomes measurable when evaluating the localization of non-palpable breast carcinoma, with just a few some reporting patient-reported outcomes, such as pain and cosmesis [24,43,45,46,48,52,55,57,59]. Of those that did, the inferiority of WGL compared to other localization strategies (i.e.: ROLL, RSL, and ML) was apparent (Supplementary Appendix 1.M). Interestingly, the included RCTs place limited emphasis upon long-term oncological outcomes, such as disease recurrence and mortality, with none of the included studies reporting these outcomes. In their prospective evaluation of patients undergoing RSL and WGL to aid tumour localization [69], Fung et al. outline that there is just a 5% anticipated recurrence (i.e.: locoregional or distant) and mortality rate expected at 5-years for patients with non-palpable breast carcinoma. These excellent oncological outcomes are best achieved through the employment of multimodal strategies to establish disease control and speaks to our multidisciplinary approach to care [70]. While emphasis is placed upon measuring margin status as a valid endpoint of these studies, we must acknowledge that positive margins translate directly into increased recurrence rates leading to recommendations from SSO-ASTRO to omit re-excision in specimens with ‘no ink on tumour’ only [71,72]. Therefore, identifying strategies to facilitate the excision of clear margins is of the utmost importance to the current breast cancer paradigm.

In this study, all 24 included RCTs compared WGL with other localization techniques. As a result, 48.3% of patients had their tumours localized by WGL (2045/4236). This is unsurprising; at present, WGL is the most robustly used method of tumour localization in clinical practice [73], therefore, it is appropriate that RCTs compare novel strategies to WGL. However, there are several disadvantages of WGL. WGL adds complexity to breast surgery as the guidewires must be inserted on the morning of the procedure [11], which may reduce patient flow within the operating room. Furthermore, the introduction of the guidewire into the breast poses the inherent risk of infection, bleeding, damage to local structures [74], and has been reported to be associated with increased pain and psychological distress for patients [8]. In spite of this, WGL was not shown to be associated with increased rates of haematoma, seroma, SSIs, or poorer satisfaction rates in this NMA when compared to other localization methods. Nonetheless, this analysis successfully challenges the widespread acceptance of WGL for localization of non-palpable breast tumours on account of to the poorer oncological and surgical outcomes (i.e.: margin positivity, reoperation, etc.) observed from the pooled data from the 24 included prospective RCTs.

Despite several strengths, the authors acknowledge certain unavoidable limitations to this NMA: The paucity of randomized patients reporting surgical outcomes in relation to USGL, IL, ML, AGL and SMRI is potentially too small to draw definitive (or significant) results, which may limit the conclusions in relation to these methods of tumour localization. This may inherently lead to the potential underestimation of their true value and/or risk profile in the clinical setting. Qualitative assessment of the definitions for margin positivity vary greatly in this systematic review (Supplementary Appendix 1.C); despite current best practice guidelines being in accordance to the SSO-ASTRO consensus [71], the heterogeneity of these results add inconsistencies to data pertaining to margin status. However, all 24 RCTs measured the margin status consistently for each localization modality versus WGL, providing some degree of congruency among their own results. Furthermore, analyses of secondary outcomes (i.e.: complication, haematoma, seroma, and SSI rates) were limited to just 4 localization strategies, with reported outcomes ranging from 24.4% to 28.7% of patients in relation to seroma (1033/4236) and haematoma (1217/4236) respectively. This limits the robustness of these results. Moreover, 22.3% of the tumours included by Struik et al. were palpable (n = 15) [45]. Unfortunately, we were unable to decipher data for non-palpable or non-invasive cancers only, limiting the transferability of these findings to non-palpable cancers exclusively. As observed in the majority of RCTs in the field of surgery, none of the RCTs included in this analysis were ‘blinded’. The inability to blind surgeons to interventions leads to these RCTs to be classed as ‘open label’, making them subject to unintentional bias [75]. Potential confounders (i.e.: patient age, body mass indices, smoking habits, surgeons experience, etc.) which may influence our primary and secondary outcomes of interest have not been considered in this study. Despite these limitations, this NMA provides comprehensive analyses of available RCT data comparing methods of localizing non-palpable breast cancers for BCS.

In conclusion, this systematic review and NMA of 24 RCTs highlights that WGL and CAL have the highest margin positivity and reoperation rates for non-palpable, breast tumours, when compared to other tumour localization techniques. Novel localization methods, such as USGL, AGL, and ML, are therefore non-inferior to WGL, and provide several advantages, such as reduced margin positivity, reoperation, and patient satisfaction rates. However, caution must be taken when interpreting these results in relation to AGL and ML, as these techniques reporting the lowest rates of margin positivity may be limited due to the small sample sizes available for analysis. Thus, it is imperative these localization strategies are evaluated in the next generation of phase III, randomized studies to validate these findings and truly decipher the optimal strategy for the localization of non-palpable breast tumours.

## Author contributions

“Matthew G. Davey, Aoife J. Lowery, and Michael J. Kerin contributed to the study conception and design. Search strategy, data collection, material preparation, and data analysis were performed by Matthew G. Davey, John Phineas O'Donnell, Michael Boland and Eanna Ryan. The first draft of the manuscript was written by Matthew G. Davey under the close supervision of Aoife J. Lowery and Michael J. Kerin. All authors commented on previous versions of the manuscript and contributed to redrafting the manuscript. All authors read and approved the final manuscript. Final sign off was provided from the senior author, Aoife J. Lowery.”

## Data availability

Study data will be made available upon reasonable request from the corresponding author.

## Sources of funding

MGD received funding from the National Breast Cancer Research Institute, Ireland.

## Declaration of competing interest

None of the authors have any conflicts of interest to disclose.
