# Supplementary material for: Optimal localization strategies for non-palpable breast cancers –A network meta-analysis of randomized controlled trials
Source: Breast. 2022 Feb 8;62:103–13. doi: 10.1016/j.breast.2022.02.004 (PMC8844725; doi:10.1016/j.breast.2022.02.004)
Supplement: Multimedia component 1 [file mmc1.docx]

**Optimal Localization Strategies for Non-Palpable Breast Cancers –A Network Meta-Analysis of Randomized Controlled Trials**

**SUPPLEMENTARY APPENDICES**

**Supplementary Appendix 1.A.**

Clinicopathological characteristics of the included patients in this systematic review and network meta-analysis of 24 randomized controlled trials.

| *Parameter* | *Number (%)* |
| --- | --- |
| Mean age (range) | 56.6 (25-81) |
| Pre/peri-menopausal  Post-menopausal | 225 (19.5%)  928 (81.5%) |
| Mean tumour size (range) | 12.4mm (1-60mm) |
| Tis/T1  T2  T3 | 1381 (90.4%)  144 (9.4%)  3 (0.2%) |
| Invasive cancer  Non-invasive cancers | 1614 (63.1%)  945 (36.9%) |
| Invasive ductal carcinoma  Invasive ductal carcinoma  Other histological subtypes  DCIS/benign | 1,534 (56.0%)  170 (6.2%)  92 (3.4%)  945 (34.5%) |
| Grade 1  Grade 2  Grade 3 | 678 (33.0%)  1001 (48.8%)  373 (18.2%) |
| Lymph node negative  Lymph node positive | 2112 (85.5%)  358 (14.5%) |
| Estrogen receptor positivity  Estrogen receptor negativity | 1842 (85.0%)  325 (15.0%) |
| Progesterone receptor positivity  Progesterone receptor negativity | 823 (68.6%)  376 (32.4%) |
| HER2 positivity  HER2 negativity | 225 (14.4%)  1517 (85.6%) |

Tis; in-situ disease in the breast, T; tumour stage HER2;

human epidermal growth factor receptor-2.

**Supplementary Appendix 1.B.**

Table and plot representing the ranked strategies for breast tumour localization in reducing margin positivity, expressed as odds ratios.

| *Intervention* | *Rank 1* | *Rank 2* | *Rank 3* | *Rank 4* | *Rank 5* | *Rank 6* | *Rank 7* | *Rank 8* | *Rank 9* |
| --- | --- | --- | --- | --- | --- | --- | --- | --- | --- |
| USGL | 0.2487 | 0.33786 | 0.248663 | 0.12248 | 0.03305 | 0.006163 | 0.0021 | 0.00081 | 0.00018 |
| AGL | 0.226425 | 0.22064 | 0.215888 | 0.16803 | 0.085013 | 0.034025 | 0.02311 | 0.01486 | 0.01201 |
| IL | 0.249088 | 0.19805 | 0.191275 | 0.1612 | 0.089875 | 0.0409 | 0.02801 | 0.01975 | 0.02185 |
| ML | 0.2309 | 0.14678 | 0.152438 | 0.15313 | 0.103313 | 0.054738 | 0.04791 | 0.04166 | 0.06914 |
| SMRI | 0.04245 | 0.08505 | 0.14275 | 0.2135 | 0.217275 | 0.094913 | 0.0761 | 0.05984 | 0.06813 |
| CAL | 0.0021 | 0.00746 | 0.019763 | 0.04765 | 0.093363 | 0.125513 | 0.14981 | 0.20075 | 0.35359 |
| ROLL | 0.00015 | 0.0022 | 0.015663 | 0.06981 | 0.196038 | 0.31675 | 0.27301 | 0.10959 | 0.01679 |
| RSL | 0.000188 | 0.00196 | 0.013563 | 0.064 | 0.180138 | 0.309313 | 0.2794 | 0.12431 | 0.02713 |
| WGL | 0 | 0 | 0 | 0.00021 | 0.001938 | 0.017688 | 0.12054 | 0.42843 | 0.4312 |

WGL; wire-guided localization, USGL; ultrasound-guided localization, ROLL; radio-guided occult lesion localization, RSL; radioactive seed localization, ML; magnetic-marker localization, IL; indocyanine green fluorescence-guided lumpectomy, CAL; cryo-assisted localization, AGL; anchor-guided localization, SMRI; intraoperative supine magnetic resonance imaging.


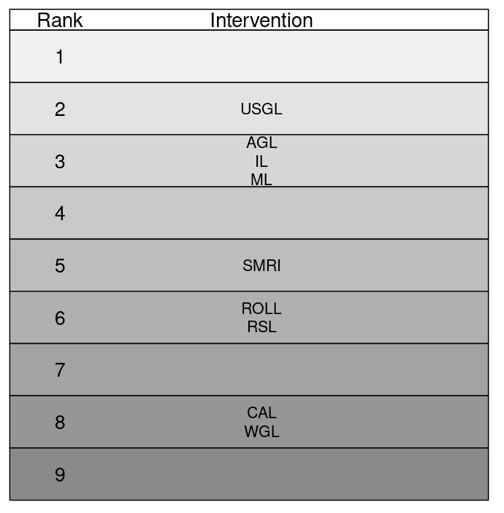


**Supplementary Appendix 1.C.**

Definitions of margin positivity in the 24 included randomized controlled trial in this study.

| *Author* | *Year* | *Definition of free/involved margins* |
| --- | --- | --- |
| Postma | 2012 | Complete removal of the tumour with tumour-free margins (i.e. no invasive carcinoma or DCIS at the inked margins). For those with free margins, the distance (mm) from the cancer cells to the nearest inked margin of the specimen was recorded. |
| Duarte | 2016 | N/R |
| Medina-Franco | 2008 | N/R |
| Rampaul | 2004 | N/R |
| Martinez | 2009 | A margin of at least 1 cm between the border of the lesion and the border of the specimen in both the pathologic and the radiologic examinations |
| Ocal | 2011 | Clearance margins >1 mm and >5 mm were accepted for invasive cancer and DCIS respectively |
| Moreno | 2008 | Clearance margin was considered as ≥ 10 mm for invasive cancer, ≥ 5 mm for ductal carcinoma in situ, and ≥ 1 mm for benign disease |
| Kanat | 2016 | N/R |
| Alikhassi | 2016 | A distance of at least 1mm between the border of the benign lesion and the boundary of the specimen upon pathologic and the radiologic examination |
| Tang | 2011 | Margins of at least 2 mm of normal breast tissue surrounding the invasive carcinoma or ductal carcinoma in-situ on final histology |
| Langhans | 2017 | Tumor cells either invasive or as DCIS were found less than 2mm from the inked margin on the microscopic examination |
| Taylor | 2021 | N/R |
| Bloomquist | 2015 | Margins were considered positive when tumour was present at the inked margin |
| Lovrics | 2011 | Margins were considered ‘‘positive’’ when gross or microscopic disease was seen at the inked margin and ‘‘close’’ when cancer cells were found less than 1 mm from the inked margin |
| Parvez | 2014 | The Prescence of tumour cells located within 1 mm from the inked margin |
| Gray | 2001 | All margins were marked with ink |
| Rahusen | 2002 | Adequate margin was defined as >1 mm |
| Hoffman | 2019 | R0 was achieved if there was no ink on the tumour during pathological workup and if tumor-free margins measured less than 1mm |
| Hu | 2020 | The surgical margins were evaluated based on current Dutch breast cancer guidelines: negative, margin distance of >4 mm; positive, margin distance of <4mm or tumor cells present at the inked edge of the specimen. |
| Tafra | 2016 | Any cancer 1 mm or less from any specimen edge |
| Struik | 2021 | Margin status was scored as the most unfavourable margin of invasive cancer and/or DCIS component, defined as (1) free, with minimal margin ≥2 mm; (2) close, with minimal margin <2 mm; (3) focally positive margin (< 4mm cumulative positive margin); or 4) more than focally positive margin |
| Tong | 2019 | Resection margins were defined with fluorescence imaging |
| Israel | 2002 | Tumor within 1 mm of the final surgical margin or the need for an additional re-excision operation, or both |
| Barth Jr. | 2019 | If invasive cancer or DCIS cells were present at the edge (on ink) or if DCIS was present less than 1 mm from the inked edge |

N/R; not reported, DCIS; ductal carcinoma in-situ, R0; complete resection

**Supplementary Appendix 1.D.**

Table and plot representing the ranked strategies for breast tumour localization in reducing the requirement for reoperation, expressed as odds ratios.

| *Intervention* | Rank 1 | Rank 2 | Rank 3 | Rank 4 | Rank 5 | Rank 6 | Rank 7 |
| --- | --- | --- | --- | --- | --- | --- | --- |
| USGL | 0.6649125 | 0.2818625 | 0.041375 | 0.0076375 | 0.002625 | 0.0010875 | 5.00E-04 |
| ML | 0.2528625 | 0.1956375 | 0.116575 | 0.0602625 | 0.054675 | 0.0572 | 0.2627875 |
| SMRI | 0.0677125 | 0.322 | 0.2839 | 0.1206625 | 0.08195 | 0.0602875 | 0.0634875 |
| ROLL | 0.00375 | 0.068175 | 0.2019625 | 0.2838875 | 0.2396625 | 0.139775 | 0.0627875 |
| CAL | 0.00645 | 0.04925 | 0.11485 | 0.1674875 | 0.1834375 | 0.2199125 | 0.2586125 |
| RSL | 0.0043125 | 0.0828625 | 0.236275 | 0.3112625 | 0.2279125 | 0.1015125 | 0.0358625 |
| WGL | 0 | 0.0002125 | 0.0050625 | 0.0488 | 0.2097375 | 0.420225 | 0.3159625 |

WGL; wire-guided localization, USGL; ultrasound-guided localization, ROLL; radio-guided occult lesion localization,

RSL; radioactive seed localization, ML; magnetic-marker localization, CAL; cryo-assisted localization, SMRI; intraoperative

supine magnetic resonance imaging.


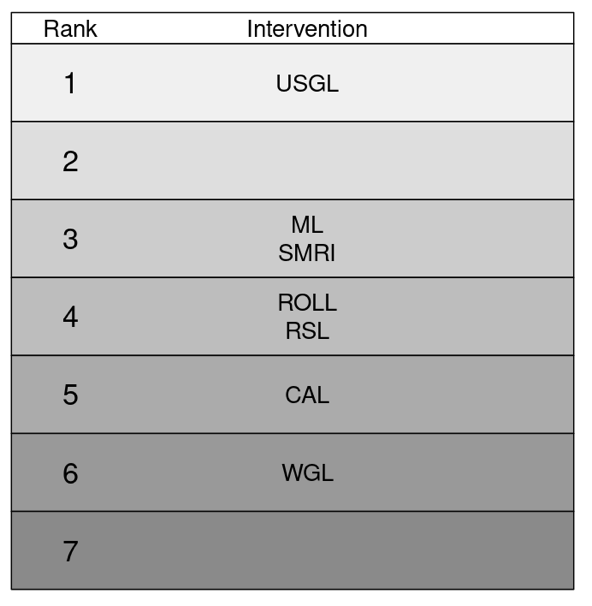


**Supplementary Appendix 1.E.**

Table and plot representing the ranked strategies for breast tumour localization methods and the duration of the procedure, expressed as odds ratios.

| *Intervention* | *Rank 1* | *Rank 2* | *Rank 3* | *Rank 4* | *Rank 5* | *Rank 6* |
| --- | --- | --- | --- | --- | --- | --- |
| RSL | 0.4525375 | 0.2085125 | 0.1224 | 0.0824125 | 0.0700625 | 0.064075 |
| IL | 0.1543875 | 0.1829125 | 0.1650625 | 0.148725 | 0.163275 | 0.1856375 |
| ROLL | 0.086825 | 0.2504875 | 0.3204625 | 0.2219875 | 0.092075 | 0.0281625 |
| USGL | 0.2081125 | 0.2288375 | 0.1786875 | 0.1365375 | 0.1303375 | 0.1174875 |
| ML | 0.09645 | 0.110325 | 0.10685 | 0.1041875 | 0.1446375 | 0.43755 |
| WGL | 0.0016875 | 0.018925 | 0.1065375 | 0.30615 | 0.3996125 | 0.1670875 |

IL; indocyanine green fluorescence-guided lumpectomy, ML; magnetic-marker localization, ROLL; radio-guided occult lesion

localization, RSL; radioactive seed localization, USGL; ultrasound guided localization, WGL; wire-guided localization.


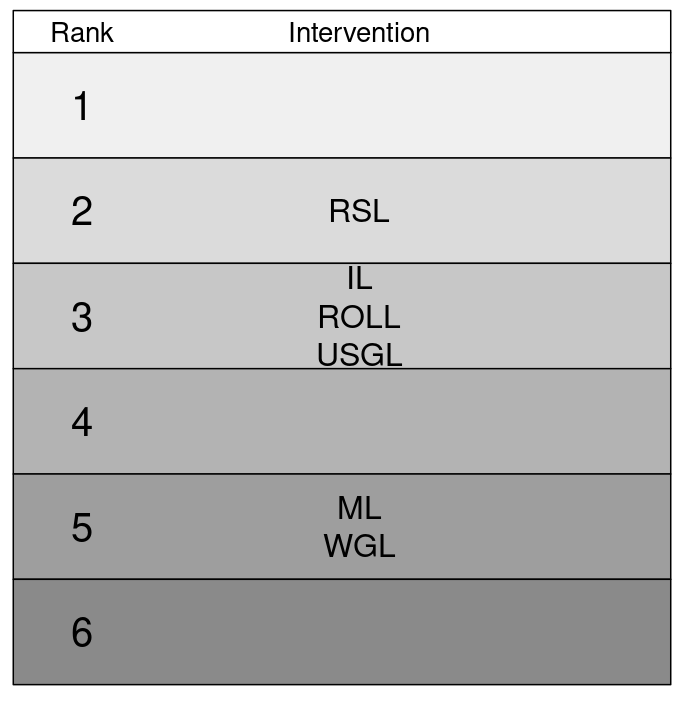


**Supplementary Appendix 1.F.**

Table representing the treatment effect strategies for breast tumour localization in (A) reducing margin positivity, (B) preventing reoperation, and (C) operation time.

| (A) | AGL | CAL | IL | ML | ROLL | RSL | SMRI | USGL | WGL |
| --- | --- | --- | --- | --- | --- | --- | --- | --- | --- |
| AGL | AGL | 3.91 (0.66, 25.14) | 1.01 (0.11, 8.85) | 1.22 (0.09, 13.44) | 2.89 (0.63, 13.85) | 2.95 (0.65, 14.4) | 1.88 (0.26, 13.95) | 0.84 (0.16, 4.66) | 4.36 (1.07, 19.96) |
| CAL | 0.26 (0.04, 1.52) | CAL | 0.26 (0.03, 1.75) | 0.31 (0.03, 2.75) | 0.75 (0.21, 2.33) | 0.76 (0.22, 2.49) | 0.48 (0.08, 2.74) | 0.22 (0.05, 0.86) | 1.12 (0.37, 3.35) |
| IL | 0.99 (0.11, 9.1) | 3.83 (0.57, 29.75) | IL | 1.2 (0.08, 15.41) | 2.85 (0.54, 16.76) | 2.92 (0.55, 17.22) | 1.86 (0.23, 16.26) | 0.82 (0.14, 5.66) | 4.31 (0.9, 24.32) |
| ML | 0.82 (0.07, 11.61) | 3.22 (0.36, 37.64) | 0.83 (0.06, 12.78) | ML | 2.38 (0.33, 22.88) | 2.44 (0.34, 23.31) | 1.56 (0.15, 20.82) | 0.69 (0.09, 7.41) | 3.59 (0.55, 33.25) |
| ROLL | 0.35 (0.07, 1.59) | 1.34 (0.43, 4.67) | 0.35 (0.06, 1.85) | 0.42 (0.04, 2.99) | ROLL | 1.02 (0.52, 2.09) | 0.65 (0.16, 2.75) | 0.29 (0.11, 0.81) | 1.51 (0.97, 2.54) |
| RSL | 0.34 (0.07, 1.55) | 1.31 (0.4, 4.5) | 0.34 (0.06, 1.8) | 0.41 (0.04, 2.92) | 0.98 (0.48, 1.92) | RSL | 0.64 (0.15, 2.69) | 0.28 (0.1, 0.79) | 1.48 (0.91, 2.52) |
| SMRI | 0.53 (0.07, 3.82) | 2.07 (0.37, 11.89) | 0.54 (0.06, 4.4) | 0.64 (0.05, 6.47) | 1.54 (0.36, 6.28) | 1.57 (0.37, 6.61) | SMRI | 0.45 (0.09, 2.26) | 2.33 (0.61, 9.07) |
| USGL | 1.19 (0.21, 6.37) | 4.65 (1.16, 19.02) | 1.21 (0.18, 7.35) | 1.45 (0.13, 11.73) | 3.47 (1.24, 9.21) | 3.53 (1.27, 9.62) | 2.24 (0.44, 11.08) | USGL | 5.22 (2.22, 12.66) |
| WGL | 0.23 (0.05, 0.94) | 0.89 (0.3, 2.67) | 0.23 (0.04, 1.11) | 0.28 (0.03, 1.82) | 0.66 (0.39, 1.03) | 0.68 (0.4, 1.1) | 0.43 (0.11, 1.63) | 0.19 (0.08, 0.45) | WGL |

| (B) | CAL | ML | ROLL | RSL | SMRI | USGL | WGL |
| --- | --- | --- | --- | --- | --- | --- | --- |
| CAL | CAL | 0.55 (0.02, 9.33) | 0.82 (0.23, 2.6) | 0.78 (0.22, 2.55) | 0.54 (0.1, 2.69) | 0.21 (0.05, 0.79) | 1.14 (0.41, 3.22) |
| ML | 1.82 (0.11, 65.1) | ML | 1.44 (0.1, 49.41) | 1.41 (0.09, 47.04) | 0.97 (0.05, 38.22) | 0.37 (0.02, 13.03) | 2.05 (0.15, 66.81) |
| ROLL | 1.23 (0.38, 4.41) | 0.69 (0.02, 10.16) | ROLL | 0.95 (0.39, 2.48) | 0.67 (0.17, 2.86) | 0.25 (0.08, 0.81) | 1.4 (0.75, 2.88) |
| RSL | 1.29 (0.39, 4.5) | 0.71 (0.02, 10.6) | 1.05 (0.4, 2.55) | RSL | 0.7 (0.17, 2.93) | 0.27 (0.08, 0.8) | 1.46 (0.79, 2.94) |
| SMRI | 1.84 (0.37, 9.55) | 1.03 (0.03, 18.57) | 1.5 (0.35, 6.05) | 1.43 (0.34, 5.97) | SMRI | 0.38 (0.08, 1.78) | 2.08 (0.61, 7.8) |
| USGL | 4.87 (1.27, 20.3) | 2.68 (0.08, 44.25) | 3.94 (1.23, 12.23) | 3.73 (1.25, 11.88) | 2.64 (0.56, 12.86) | USGL | 5.49 (2.3, 14.58) |
| WGL | 0.88 (0.31, 2.47) | 0.49 (0.01, 6.47) | 0.72 (0.35, 1.33) | 0.68 (0.34, 1.26) | 0.48 (0.13, 1.65) | 0.18 (0.07, 0.43) | WGL |

| (C) | IL | ML | ROLL | RSL | USGL | WGL |
| --- | --- | --- | --- | --- | --- | --- |
| IL | IL | 2.95 (-15.54, 21.35) | -0.84 (-14.26, 12.31) | -4 (-21.84, 13.65) | -1.28 (-17.06, 15.17) | 2 (-10.33, 14.21) |
| ML | -2.95 (-21.35, 15.54) | ML | -3.84 (-18.75, 11.08) | -7.01 (-25.78, 11.79) | -4.25 (-21.32, 13.51) | -1 (-14.92, 13.07) |
| ROLL | 0.84 (-12.31, 14.26) | 3.84 (-11.08, 18.75) | ROLL | -3.16 (-16.81, 10.62) | -0.39 (-11.73, 11.65) | 2.86 (-2.29, 8.1) |
| RSL | 4 (-13.65, 21.84) | 7.01 (-11.79, 25.78) | 3.16 (-10.62, 16.81) | RSL | 2.73 (-13.38, 19.62) | 5.99 (-6.74, 18.71) |
| USGL | 1.28 (-15.17, 17.06) | 4.25 (-13.51, 21.32) | 0.39 (-11.65, 11.73) | -2.73 (-19.62, 13.38) | USGL | 3.25 (-7.6, 13.4) |
| WGL | -2 (-14.21, 10.33) | 1 (-13.07, 14.92) | -2.86 (-8.1, 2.29) | -5.99 (-18.71, 6.74) | -3.25 (-13.4, 7.6) | WGL |

IL; indocyanine green fluorescence-guided lumpectomy, ML; magnetic-marker localization,

ROLL; radio-guided occult lesion localization, RSL; radioactive seed localization, USGL;

ultrasound guided localization, WGL; wire-guided localization.

**Supplementary Appendix 1.G.**

Table and plot representing the ranked strategies for breast tumour localization and complication rates, expressed as odds ratios.

| *Intervention* | *Rank 1* | *Rank 2* | *Rank 3* | *Rank 4* |
| --- | --- | --- | --- | --- |
| ML | 0.529775 | 0.1297875 | 0.10125 | 0.2391875 |
| ROLL | 0.304675 | 0.4140125 | 0.1800875 | 0.101225 |
| RSL | 0.123675 | 0.194775 | 0.238525 | 0.443025 |
| WGL | 0.041875 | 0.261425 | 0.4801375 | 0.2165625 |

ML; magnetic-marker localization, ROLL; radio-guided occult lesion localization, RSL;

radioactive seed localization, WGL; wire-guided localization.


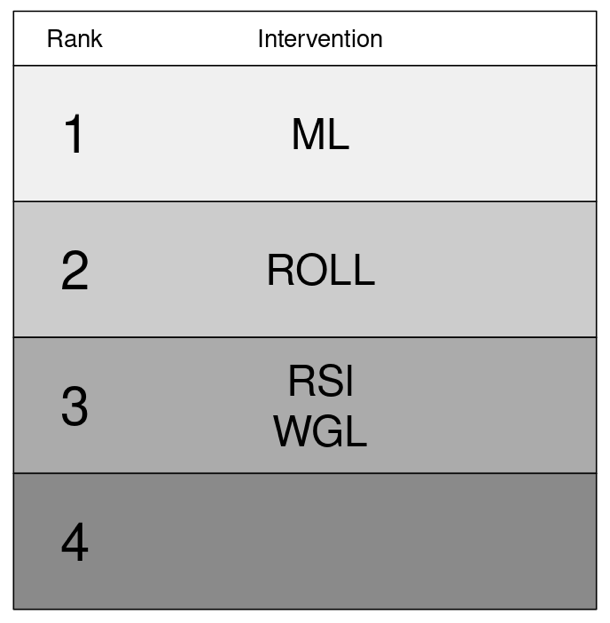


**Supplementary Appendix 1.H.**

Table and plot representing the ranked strategies for breast tumour localization and haematoma rates, expressed as odds ratios.

| *Intervention* | *Rank 1* | *Rank 2* | *Rank 3* | *Rank 4* |
| --- | --- | --- | --- | --- |
| RSL | 0.3589125 | 0.319475 | 0.154575 | 0.1670375 |
| ML | 0.5342 | 0.2036875 | 0.114125 | 0.1479875 |
| WGL | 0.07245 | 0.3646625 | 0.4755625 | 0.087325 |
| ROLL | 0.0344375 | 0.112175 | 0.2557375 | 0.59765 |

ML; magnetic-marker localization, WGL; wire-guided localization. RSL; radioactive seed

localization, ROLL; radio-guided occult lesion localization


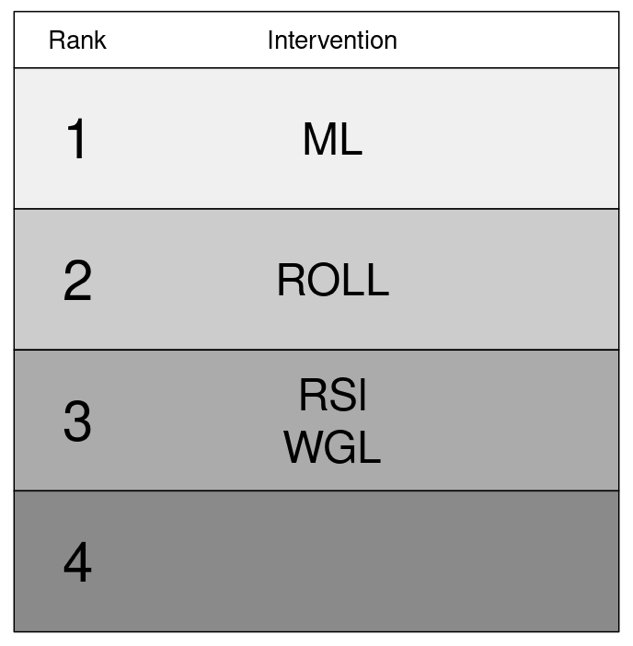


**Supplementary Appendix 1.I.**

Table and plot representing the ranked strategies for breast tumour localization and seroma rates, expressed as odds ratios.

| *Intervention* | *Rank 1* | *Rank 2* | *Rank 3* | *Rank 4* |
| --- | --- | --- | --- | --- |
| ROLL | 0.7262 | 0.20465 | 0.0581125 | 0.0110375 |
| ML | 0.1320625 | 0.115125 | 0.2734625 | 0.47935 |
| RSL | 0.0620125 | 0.10205 | 0.3688875 | 0.46705 |
| WGL | 0.079725 | 0.578175 | 0.2995375 | 0.0425625 |

ROLL; radio-guided occult lesion localization, WGL; wire-guided localization, ML;

magnetic-marker localization, RSL; radioactive seed localization


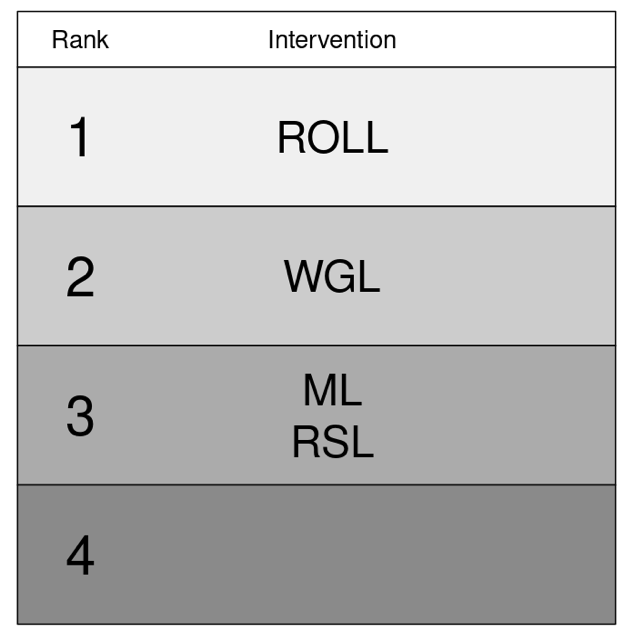


**Supplementary Appendix 1.J.**

Table and plot representing the ranked strategies for breast tumour localization and surgical site infection rates, expressed as odds ratios.

| *Intervention* | *Rank 1* | *Rank 2* | *Rank 3* | *Rank 4* |
| --- | --- | --- | --- | --- |
| ROLL | 0.631775 | 0.250025 | 0.1148 | 0.0034 |
| RSL | 0.2722625 | 0.2461 | 0.4612 | 0.0204375 |
| WGL | 0.0833875 | 0.4948125 | 0.4117625 | 0.0100375 |
| ML | 0.012575 | 0.0090625 | 0.0122375 | 0.966125 |

ROLL; radio-guided occult lesion localization, RSL; radioactive seed localization, WGL;

wire-guided localization, ML; magnetic-marker localization


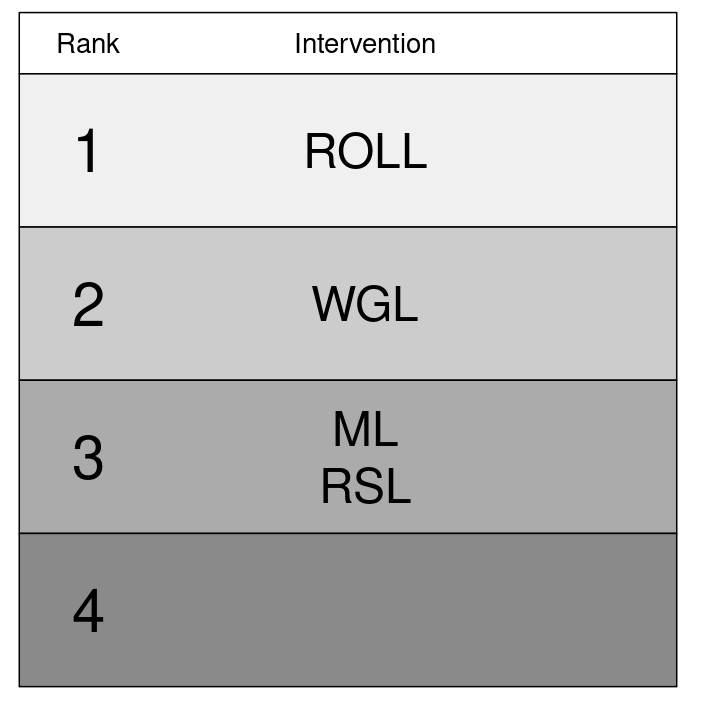


**Supplementary Appendix 1.K.**

Table representing the treatment effect strategies for breast tumour localization in (A) overall complications, (B) haematoma, (C) seroma, and (D) surgical site infections.

| (A) | ML | ROLL | RSl | WGL |
| --- | --- | --- | --- | --- |
| ML | ML | 1.45 (0.03, 108.6) | 2.66 (0.04, 237.79) | 2.3 (0.07, 140.22) |
| ROLL | 0.69 (0.01, 29.29) | ROLL | 1.86 (0.15, 18.11) | 1.6 (0.39, 5.96) |
| RSl | 0.38 (0, 22.86) | 0.54 (0.06, 6.69) | RSL | 0.85 (0.13, 6.68) |
| WGL | 0.44 (0.01, 15.06) | 0.63 (0.17, 2.54) | 1.18 (0.15, 7.69) | WGL |

| (B) | ML | ROLL | RSL | WGL |
| --- | --- | --- | --- | --- |
| ML | ML | 4.11 (0.19, 197.42) | 1.57 (0.04, 104.38) | 2.34 (0.14, 106.6) |
| ROLL | 0.24 (0.01, 5.32) | ROLL | 0.37 (0.03, 4.28) | 0.59 (0.17, 1.84) |
| RSL | 0.64 (0.01, 27.01) | 2.67 (0.23, 36.19) | RSL | 1.54 (0.18, 16.36) |
| WGL | 0.43 (0.01, 7.03) | 1.69 (0.54, 5.98) | 0.65 (0.06, 5.46) | WGL |

| (C) | ML | ROLL | RSL | WGL |
| --- | --- | --- | --- | --- |
| ML | ML | 0.17 (0, 4.91) | 1 (0.01, 58.92) | 0.35 (0.01, 8.34) |
| ROLL | 5.94 (0.2, 367.47) | ROLL | 5.79 (0.45, 135.86) | 2.03 (0.61, 10.07) |
| RSL | 1 (0.02, 87.52) | 0.17 (0.01, 2.22) | RSL | 0.36 (0.02, 3.79) |
| WGL | 2.85 (0.12, 124.06) | 0.49 (0.1, 1.64) | 2.81 (0.26, 42.97) | WGL |

| (D) | ML | ROLL | RSL | WGL |
| --- | --- | --- | --- | --- |
| ML | ML | 0 (0, 0.46) | 0 (0, 1.25) | 0 (0, 0.97) |
| ROLL | 320020133.78 (2.17, 1.89341854990882e+28) | ROLL | 2.04 (0.11, 91.34) | 2.08 (0.46, 21.38) |
| RSL | 135110197.18 (0.8, 7.8124470425573e+27) | 0.49 (0.01, 8.92) | RSL | 1 (0.06, 15.78) |
| WGL | 134310947.12 (1.03, 7.1055150552816e+27) | 0.48 (0.05, 2.17) | 1 (0.06, 15.78) | WGL |

ML; magnetic-marker localization, ROLL; radio-guided occult lesion localization, RSL; radioactive seed localization, WGL; wire-guided localization.

**Supplementary Appendix 1.L.**

Forest plots comparing wire-guided localization (WGL) to magnetic-marker localization (ML), radio-guided occult lesion localization (ROLL) and radioactive seed localizer (RSL) for (A) patient dissatisfaction, and (B) surgeon dissatisfaction, as appropriate.

A


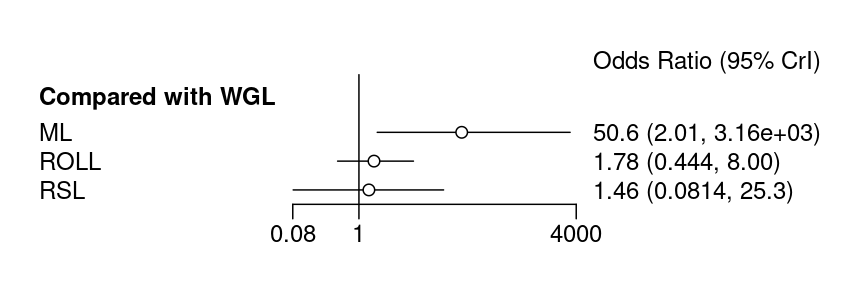


B


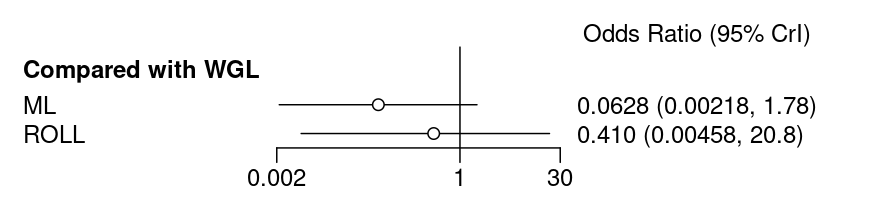


**Supplementary Appendix 1.M.**

Table illustrating patient reported outcome measures discomfort and cosmesis.

| *Author* | *Year* | *Total (N)* | *Breast (N)* | *Intervention (vs. WGL)* | *Pain Measurement Scale* | *Pain - Intervention (vs. WGL) (median, (IQR)/Mean(SD))* | *Cosmesis Measurement Scale* | *Patient Cosmesis - Intervention (vs. WGL) (mean, standard deviation)* |
| --- | --- | --- | --- | --- | --- | --- | --- | --- |
| Postma | 2012 | 314 | 316 | ROLL | VAS | 3 (1.0 – 10.0) vs. 4 (1.0 – 10.0) | Likert | 8 (1.0 – 10.0) vs. 8 (1.0 – 10.0) |
| Duarte | 2016 | 129 | 129 | ROLL | VAS | 3.0 (2.0) vs. 3.7 (2.1) | N/R | - |
| Medina-Franco | 2008 | 100 | 100 | ROLL | N/R | - | Four-point | Excellent: 38/50 vs. 26/50 |
| Rampaul | 2004 | 93 | 93 | ROLL | Likert | 2.7 vs. 3.6 | N/R | - |
| Martinez | 2009 | 134 | 134 | ROLL | N/R | - | N/R | - |
| Ocal | 2011 | 108 | 108 | ROLL | N/R | - | N/R | - |
| Moreno | 2008 | 120 | 120 | ROLL | VAS | 1.6 vs. 2.2 | Three-point | Excellent: 57/61 vs. 49/59 |
| Kanat | 2016 | 36 | 36 | ROLL | VAS | 2.0 (0.8) vs. 2.6 (0.7) | VAS | 8.2 (1.2) vs. 7.0 (1.0) |
| Alikhassi* | 2016 | 60 | 60 | ROLL | N/R | - | N/R | - |
| Tang | 2011 | 157 | 157 | ROLL + Dye | N/R | - | N/R | - |
| Langhans | 2017 | 409 | 413 | RSL | VAS | - | N/R | - |
| Taylor | 2021 | 659 | 664 | RSL | VAS | - | N/R | - |
| Bloomquist | 2015 | 125 | 125 | RSL | Likert | 2.0 (1.0 – 5.0) vs. 2.0 (1.0 – 5.0) | N/R | - |
| Lovrics | 2011 | 305 | 305 | RSL | Likert | - | N/R | - |
| Parvez | 2014 | 73 | 73 | RSL | N/R | - | EORTC | 6.0 (4.0) vs. 5.5 (3.4) |
| Gray | 2001 | 97 | 97 | RSL | N/R | - | N/R | - |
| Rahusen | 2002 | 49 | 49 | USGL | N/R | - | N/R | - |
| Hoffman | 2019 | 47 | 47 | USGL | N/R | - | N/R | - |
| Hu | 2020 | 520 | 520 | USGL | N/R | - | N/R | - |
| Tafra | 2016 | 320 | 320 | CAL | N/R | - | N/R | - |
| Struik | 2021 | 67 | 67 | ML | VAS | 3.0 (3.0 – 3.0) vs. 7 (4.5 – 8.0) | N/R | - |
| Tong | 2019 | 62 | 62 | IL | N/R | - | N/R | - |
| Israel | 2002 | 114 | 114 | AGL | N/R | - | N/R | - |
| Barth Jr. | 2019 | 137 | 137 | SMRI | N/R | - | N/R | - |

N; Number, WGL; wire-guided localization, IQR; interquartile range, ROLL; radio-guided occult lesion localization, RSL; radioactive seed localization, USGL; ultrasound-guided localization, CAL; cryo-assisted localization, ML; magnetic-marker localization, IL; indocyanine green fluorescence-guided lumpectomy, AGL; anchor-guided localization, VAS; visual analogue scale, N/R; not reported, EORTC; European Organization for Research and Treatment of Cancer

**Supplementary Appendix 1.N.**

Forest plots comparing (WGL) to radio-guided occult lesion localization (ROLL), radioactive seed localization (RSL), supine magnetic resonance imaging (SMRI), and ultrasound-guided localization (USGL) for (A) specimen size, (B) specimen volume, and (C) specimen weight, as appropriate.

A


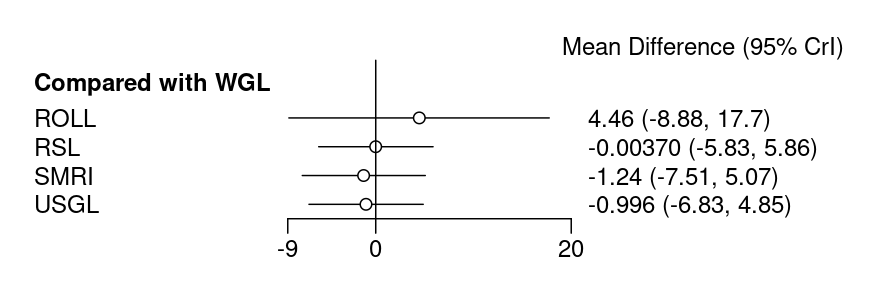


B


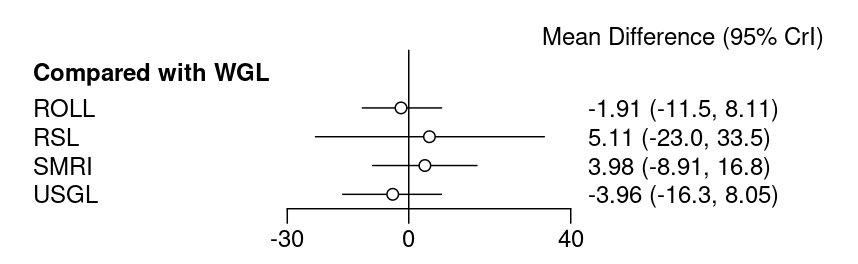


C


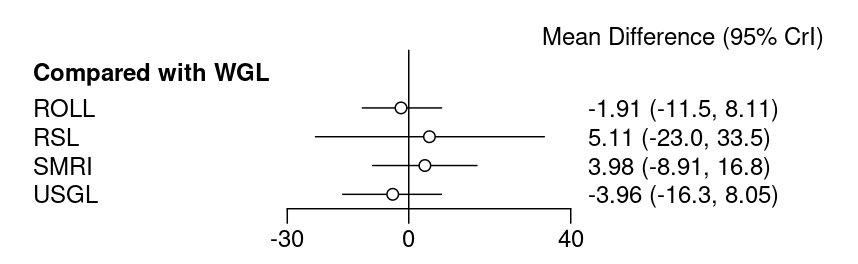


**Supplementary Appendix 1.O.**

Risk of bias assessment.


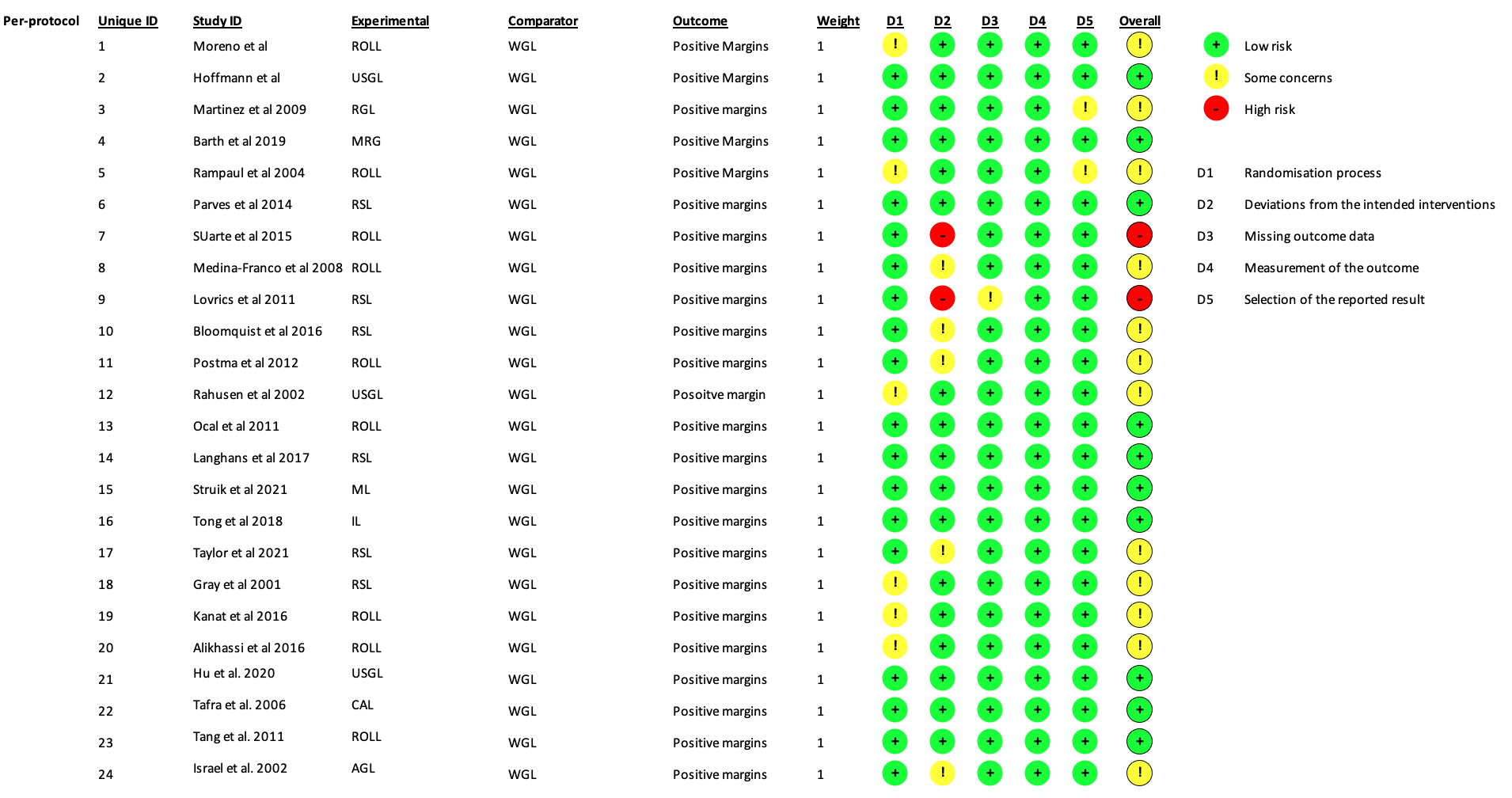


WGL; wire-guided localization, USGL; ultrasound-guided localization, ROLL; radio-guided occult lesion localization, RSL; radioactive seed localization, ML; magnetic-marker localization, IL; indocyanine green fluorescence-guided lumpectomy, CAL; cryo-assisted localization, AGL; anchor-guided localization, SMRI; intraoperative supine magnetic resonance imaging.
